# Antibiotic susceptibility, cytotoxicity, and protease activity of viridans group streptococci causing endophthalmitis

**DOI:** 10.1371/journal.pone.0209849

**Published:** 2018-12-21

**Authors:** Mary E. Marquart, Angela H. Benton, Regina C. Galloway, Lisa M. Stempak

**Affiliations:** 1 Department of Microbiology and Immunology, University of Mississippi Medical Center, Jackson, Mississippi, United States of America; 2 Department of Pathology, University of Mississippi Medical Center, Jackson, Mississippi, United States of America; University of Georgia, UNITED STATES

## Abstract

The viridans group streptococci comprise multiple species and have gained more recognition in recent years as common etiologic agents of bacterial endophthalmitis. The purpose of this study was to identify the species of human endophthalmitis isolates of viridans streptococci and to characterize their potential virulence attributes. The species of 22 endophthalmitis strains of viridans streptococci were identified by Matrix Assisted Laser Desorption Ionization Time-of-Flight. Susceptibilities to 3 antibiotics commonly used for bacterial endophthalmitis were determined. The extracellular milieu of each strain was tested for cytotoxicity of retinal pigmented epithelial cells, hemolysis of sheep erythrocytes, and protease activity using gelatin zymography. Identified species were *Streptococcus mitis/oralis*, *S*. *salivarius*, *S*. *vestibularis*, *S*. *parasanguinis*, *S*. *mutans*, *S*. *constellatus*, and *S*. *gordonii*. One strain of *S*. *pseudoporcinus* was also identified. All strains were sensitive to vancomycin, 77% were resistant to amikacin, and 27% had intermediate resistance to ceftazidime. Extracellular milieu from all strains except one (*S*. *pseudoporcinus*) were largely devoid of toxicity to retinal pigmented epithelial cells and sheep erythrocytes. Twelve strains, 10 of which were *S*. *mitis/oralis*, produced protease activity. Interestingly, not all of the *S*. *mitis/oralis* strains were proteolytic. These findings highlight the diversity of virulence factor production in ocular strains of the viridans streptococci not only at the group level but also at the species level.

## Introduction

The term “viridans group streptococci (VGS)” is often used to describe a large group of *Streptococcus* species that historically did not fit into the Lancefield typing scheme. Classification of members of this group has been problematic due to misidentification and the wide genetic heterogeneity among species [1–4]. The VGS have been identified as mostly exhibiting alpha-hemolysis on blood agar similar to *Streptococcus pneumoniae*, but having resistance to optochin and insolubility in bile salts which are opposite characteristics of *S*. *pneumoniae*. However, there exist exceptions to these generalizations, as some VGS exhibit beta- or gamma-hemolysis and/or are sensitive to optochin or bile. Further complicating classification is the reactivity of some species to Lancefield sera. This overarching collection of VGS is divided into 5 major groups (mitis, anginosus, sanguinis, salivarius, and mutans) each consisting of multiple different species [2]. Many species from these groups are commensals of the human oral cavity.

VGS have been emerging more frequently as causes of bacterial endophthalmitis in the scientific literature in recent years. Endophthalmitis cases caused by streptococcal species are associated with poor visual outcomes such as loss of light perception, evisceration, and enucleation [5–11]. In many cases, the VGS are the second most commonly identified causative agents of bacterial endophthalmitis after coagulase-negative staphylococci [8,12–16]. Although the incidence of bacterial endophthalmitis is low, ocular surgeries and intravitreal injections of therapeutic agents are increasing, and the VGS have emerged as pathogens associated with infections following intravitreal injections [6,17–19].

Previous studies have examined the pathogenesis and virulence of VGS in the context of oral disease and endocarditis (for a review, see Nakano *et al*.) [20]. VGS in the oral cavity are the early colonizers that reside in different mucosal niches as well as on the salivary pellicle of tooth surfaces to initiate biofilm formation. Their ability to attach to different receptors both on the pellicle as well as on other species has long been considered an important virulence trait for the development of caries, as caries can develop following mature biofilm formation of a complex community of bacteria. Metabolic substrate utilization is another virulence characteristic of the VGS in the oral cavity. The production of lactic acid by the VGS following fermentation of dietary sugars contributes to destruction of tooth enamel. These bacteria can enter the bloodstream of the patient during dental procedures and have a propensity for targeting patients with damaged cardiac valves. Binding to the fibroblasts and platelets of damaged valves, as well as platelet aggregation, has been proposed to be mediated by the rhamnose-glucose polysaccharide, a collagen-binding protein [20], and serine-rich repeat motif adhesins [21] located in the cell wall.

To the best of our knowledge, no information regarding pathogenic factors and mechanisms of this group of bacteria in the context of endophthalmitis has been reported. We hypothesized that, given the likely genetic heterogeneity of VGS and their natural transformation ability to exchange genes with other streptococcal species through horizontal exchange of chromosomal DNA, a collection of VGS isolated from endophthalmitis could vary in their possession of commonly suspected virulence factors. Previous work on *S*. *pneumoniae* endophthalmitis showed that the cholesterol-dependent cytolysin, pneumolysin, produced by this species is important for pathogenesis in the vitreous humor [22], and the gene encoding pneumolysin is present in some VGS [4,23]. Based on this information and on studies of other endophthalmitis pathogens producing proteases involved in virulence [24–26], we focused on determining whether toxins and proteases were produced by VGS endophthalmitis strains. We obtained 22 human endophthalmitis strains of supposed VGS and identified the species of each strain. We then screened for the most likely virulence factors (toxins and proteases) in addition to susceptibilities to 3 common antibiotics used in the treatment of bacterial endophthalmitis.

## Materials and methods

### Bacterial strains and species identification

Twenty-two human endophthalmitis strains of non-*S*. *pneumoniae*, alpha-hemolytic streptococci were kindly provided by Regis P. Kowalski (Charles T. Campbell Eye Microbiology Laboratory, Pittsburgh, PA, USA) and Darlene Miller (Bascom-Palmer, Miami, FL, USA). Each strain was received on blood agar and was immediately re-cultured for isolation of individual colonies on 5% sheep blood agar with 18–24 hour incubation at 37°C and 5% CO_2_. Isolated colonies were cultured in Todd Hewitt broth containing 0.5% yeast extract (THY) at 37°C and 5% CO_2_ and prepared as frozen stocks in 20% glycerol-containing THY.

Species were identified by Matrix Assisted Laser Desorption Ionization Time-of-Flight (MALDI-TOF) with the VITEK MS mass spectrometer according to manufacturer instructions (bioMérieux Inc., Durham, NC, USA). One strain was unable to be identified by MALDI-TOF and was instead identified by biochemical characteristics using the VITEK 2 according to the manufacturer (bioMérieux Inc.). *S*. *mitis* and *S*. *oralis* cannot currently be differentiated from each other by these methods.

### Antibiotic susceptibility determination

The susceptibility of each VGS strain to amikacin, ceftazidime, and vancomycin was tested by measurement of zones of inhibition of bacterial growth surrounding antibiotic-containing disks on blood agar. Each strain was suspended to a 0.5 McFarland standard then spread onto blood agar. Antibiotic disks (Oxoid, Basingstoke, UK) were placed on the agar prior to 18 hours of incubation at 37°C and 5% CO_2_. Performance standards for vancomycin define strains as either susceptible or resistant, but not intermediate; VGS which are inhibited at zones of ≥17 mm are considered susceptible to vancomycin [27]. Breakpoint ranges for ceftazidime and VGS are not published, therefore, the breakpoint ranges for a same-generation cephalosporin (cefotaxime) were chosen for ceftazidime. Lastly, for amikacin, there are no breakpoints for any streptococcal species, so the breakpoints for staphylococci were used [27].

### Preparation of concentrated extracellular milieu

Each strain was isolated on blood agar from frozen stock and isolated colonies were grown as starter cultures in THY for 8 hours at 37°C and 5% CO_2_. Starter cultures were then diluted 100-fold in fresh, pre-warmed THY and incubated for 16 hours. Culture purity was verified by plating on blood agar and examining colony growth. Each culture was centrifuged for 30 minutes at 4°C and 4500 rpm, and the extracellular milieu (supernatant) was passed through a 0.22 μm filter. Each filtered supernatant underwent 200-fold concentration at 4°C via a centrifugal filter device with a 10-kDa molecular mass cutoff. Concentrated supernatants were aliquoted and frozen for use in subsequent assays of function. THY alone was treated in the same manner, with incubation, filtration, and concentration, to serve as a control. *S*. *pneumoniae* D39 concentrated supernatant was also prepared to serve as a control.

Total protein concentrations of the 200-fold concentrated supernatants and concentrated THY were determined using bicinchoninic acid-based protein assays and bovine serum albumin standards. All protein samples were adjusted to 20 mg/mL with PBS, and 2-fold dilutions were prepared to yield additional samples of 10, 5, and 2.5 mg/mL. A minimum of 2 biological replicates of supernatant were prepared from each bacterial strain using separately-inoculated cultures.

### Cytotoxicity assays

Human retinal pigmented epithelial cells (ARPE-19; #CRL-2302, American Type Culture Collection, Manassas, VA, USA; purchased directly from ATCC) were cultured as monolayers in Dulbecco’s Modified Eagle’s Medium/F-12 (DME/F-12) containing 2.5 mM L-glutamine and 15 mM HEPES buffer (HyClone Laboratories, Logan, UT, USA) with 10% fetal bovine serum and cell culture grade penicillin/streptomycin. Cells were maintained at 37°C and 5% CO_2_ and split 5-fold every week. Passages 9–11 were used for cytotoxicity assays. Cells were seeded into 96-well microplates and grown to near confluency. Two to 4 hours prior to cytotoxicity assays, the monolayers were washed once with PBS and then maintained in DME/F-12 without serum or antibiotics. One well from each plate was used to quantitate cell number per well with the aid of a hemacytometer to ensure assay-to-assay consistency of cell numbers (range: 3.8 X 10^3^ to 8.4 X 10^3^ cells per well).

The cell monolayer of each well was washed twice with PBS following the incubation in non-serum, non-antibiotic-containing medium, and then 0.1 mL PBS was applied. Each bacterial supernatant or THY (0.05 mL) was applied in triplicate in concentrations of 20, 10, 5, and 2.5 mg/mL. PBS alone was applied as a negative control for cell viability, and 0.5% saponin and *S*. *pneumoniae* D39 supernatants were applied as positive controls for cell death. Cells were incubated with the samples for 2 hours at 37°C and 5% CO_2_, washed with PBS, then incubated with 2 μM calcein AM (Invitrogen Molecular Probes, Eugene, OR, USA) for 30 minutes to stain viable cells. Fluorescence intensity was measured at an excitation of 485 nm and emission at 520 nm.

Technical triplicates of at least two independent biological replicates were measured. The average of each technical triplicate was treated as one data point for a given biological sample. Spectrofluorometric values were normalized by setting the highest triplicate average of each plate to 100 using a multiplication factor and then adjusting all of the other values from the same microplate according to that multiplication factor. One-way analysis of variance was used to determine overall significance of the collective data, and then pairwise comparisons were made using a 2-tailed t-test accounting for unequal variance.

### Hemolysis assays

Lysis of sheep erythrocytes was measured by incubation of a specified range of concentrations of each supernatant with the erythrocytes followed by spectrophotometric measurement of hemoglobin release at 450 nm. Supernatants (0.05 mL) at 20, 10, 5, or 2.5 mg/mL were incubated in the presence of 0.05 mL 5% defibrinated sheep erythrocytes (washed 2 times prior to assay) and 0.1 mL PBS in microplate wells with curved bottoms for 30 minutes at 37°C and 5% CO_2_. Controls included THY incubated with the erythrocytes at the same concentrations as the supernatants, individual supernatants and PBS in the absence of erythrocytes for background subtraction, PBS and erythrocytes, 0.5% saponin (positive lysis control), and *S*. *pneumoniae* D39 supernatant (positive lysis control). Microplates were centrifuged at 3200 rpm for 5 minutes to collect non-lysed erythrocytes, then 0.1 mL of supernatant was carefully removed from each well and placed in individual wells of a flat-bottomed microplate. Absorbance was measured spectrophotometrically at 450 nm to obtain relative hemolysis values and individual backgrounds were subtracted for each well. Technical triplicates of at least two independent biological replicates were measured. The average of each technical triplicate was treated as one data point for a given biological sample. Spectrophotometric values were normalized by setting the average positive control (saponin) to 100 for each microplate using a multiplication factor and then adjusting all of the other values from the same microplate according to that multiplication factor. One-way analysis of variance was used to determine overall significance of the collective data, and then pairwise comparisons were made using a 2-tailed t-test accounting for unequal variance.

### Polymerase chain reaction

Genomic DNA from each streptococcal strain was purified by phenol:chloroform extraction of bacterial lysates collected from stationary phase cultures followed by ethanol precipitation and suspension in sterile water. Each genomic DNA preparation (200 ng) was added to 2X GoTaq (Promega, Madison, WI, USA), sterile water, and 4 μM of each of 2 primers to test for amplification of the gene encoding pneumolysin. The primer sequences were 5’-atggcaaataaagcagtaaatg-3’ and 5’-gtcattttctaccttatcctctacc-3’ and were specific for the beginning and the end of the gene for reference strain *S*. *pneumoniae* D39 (GenBank Accession # NC_008533.1). The PCR reaction consisted of an initial denaturation at 97°C for 5 minutes followed by 30 cycles of 95°C for 1 minute, 49°C for 1 minute, and 72°C for 2 minutes. A final extension of 10 minutes at 72°C was performed. The expected amplicon was 1416 base pairs. *S*. *pneumoniae* D39 and *Haemophilus influenzae* DNA (kindly provided by Sandy Wong, University of Mississippi Medical Center, Jackson, MS, USA) served as positive and negative controls, respectively. PCR was performed with at least 2 independent preparations of genomic DNA for each strain.

### Zymography

Streptococcal supernatants were tested for protease activity by gelatin zymography as described previously [28]. Each sample (0.2 mg), including THY and *S*. *pneumoniae* D39, was loaded onto a 10% SDS-polyacrylamide gel containing 0.1% gelatin. Protease bands were visualized by Coomassie counterstaining after electrophoresis and incubation for 48 hours as previously described [28]. Zymography was repeated with a second set of biological replicates.

## Results

### Species identification

Twenty-one strains were identified by MALDI-TOF and one strain (E618) was identified by biochemical characteristics (Table 1). Fifteen (68%) strains were identified as *S*. *mitis/oralis*, species that are commonly referred to as VGS or oral streptococci. The remainder of the strains were also species commonly located in the oral cavity, with the exception of E618, which was identified as *S*. *pseudoporcinus*.

**Table 1:** Species identification of clinical endophthalmitis strains.

Strain Designation	Species	Source
E618	*S*. *pseudoporcinus*	Campbell Lab
E619	*S*. *mitis/oralis*	Campbell Lab
E628	*S*. *mitis/oralis*	Campbell Lab
E636	*S*. *salivarius* ssp. *salivarius*	Campbell Lab
E647	*S*. *mitis/oralis*	Campbell Lab
E649	*S*. *mitis/oralis*	Campbell Lab
E651	*S*. *mitis/oralis*	Campbell Lab
E653	*S*. *vestibularis*	Campbell Lab
E664	*S*. *mitis/oralis*	Campbell Lab
E665	*S*. *mitis/oralis*	Campbell Lab
E669	*S*. *parasanguinis*	Campbell Lab
E681	*S*. *mutans*	Campbell Lab
E684	*S*. *mitis/oralis*	Campbell Lab
E689	*S*. *mitis/oralis*	Campbell Lab
E697	*S*. *mitis/oralis*	Campbell Lab
E707	*S*. *mitis/oralis*	Campbell Lab
E728	*S*. *mitis/oralis*	Campbell Lab
E734	*S*. *mitis/oralis*	Campbell Lab
11–4097	*S*. *mitis/oralis*	Bascom-Palmer
11–6117	*S*. *constellatus*	Bascom-Palmer
14–4065	*S*. *gordonii*	Bascom-Palmer
14–4881	*S*. *mitis/oralis*	Bascom-Palmer

### Antibiotic susceptibility

Strains were tested for susceptibility to 3 commonly used antibiotics for endophthalmitis–amikacin, ceftazidime, and vancomycin–by measurement of zones of inhibition surrounding antibiotic discs. The majority (77%) of the strains were resistant to amikacin, whereas all were susceptible to vancomycin (Table 2). Approximately 27% of the strains possessed intermediate resistance to ceftazidime.

**Table 2:** Antibiotic susceptibilities of clinical endophthalmitis strains.

Strain	Amikacin	Ceftazidime	Vancomycin
E618	S	S	S
E619	R	S	S
E628	R	S	S
E636	R	I	S
E647	S	S	S
E649	R	I	S
E651	R	I	S
E653	I	S	S
E664	R	S	S
E665	R	I	S
E669	R	S	S
E681	R	S	S
E684	R	S	S
E689	S	S	S
E697	R	S	S
E707	R	S	S
E728	R	S	S
E734	R	I	S
11–4097	R	S	S
11–6117	R	I	S
14–4065	R	S	S
14–4881	S	S	S

R, resistant; S, susceptible; I, intermediate

### Cytotoxicity

**Fig 1:**
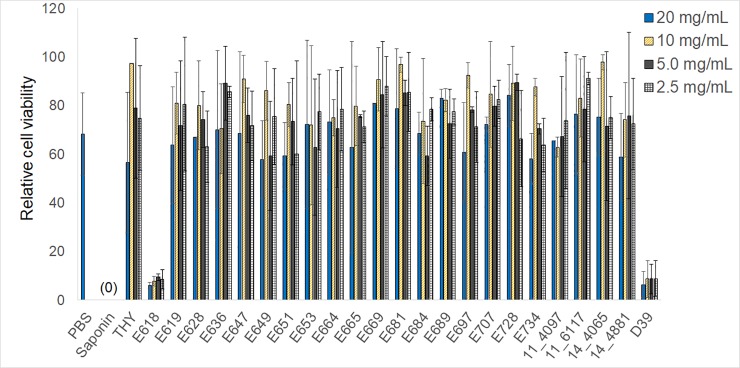
Relative mean viability of ARPE-19 cells following exposure to bacterial supernatants. Each supernatant at the indicated concentrations, in addition to PBS, THY, and saponin, is listed on the x-axis. The y-axis indicates relative viability after adjustment to a scale of 100 as described in the materials and methods. Error bars indicate standard deviation. Indicators of significant differences (described in the results) are not depicted to enhance figure clarity. Data represent the averages obtained from at least 2 independent experiments.

The extracellular milieu of each strain was examined for cytotoxicity of ARPE-19 cells since most bacterial toxins are secreted. One-way analysis of variance of controls and all samples at all concentrations indicated overall significance in relative percent cell viability (p = 0.01; Fig 1). Pairwise comparisons between each sample and PBS (negative control), saponin (positive control), and *S*. *pneumoniae* D39 (known to produce pneumolysin) determined the following: 1) THY was not significantly different than PBS or saponin at 20, 5, and 2.5 mg/mL (p > 0.13), but was significantly different from both PBS and saponin at 10 mg/mL (p < 0.01); 2) D39 was significantly different from PBS (p < 0.01) and not significantly different from saponin (p >0.29) at all concentrations; 3) Any VGS sample that was significantly different from PBS was also significantly different from saponin, except E618 at 2.5 mg/mL, which was significantly different from PBS (p < 0.01) but not saponin (p = 0.07); 4) Between 10 and 13 VGS samples, depending on the concentration, were significantly different from D39. Cells exposed to E618 supernatants at all 4 concentrations were approximately 6–9% viable, similar to those exposed to D39 at the 4 concentrations (p > 0.89). Concentration-dependent effects were not apparent at the concentrations tested.

### Hemolytic activity

**Fig 2:**
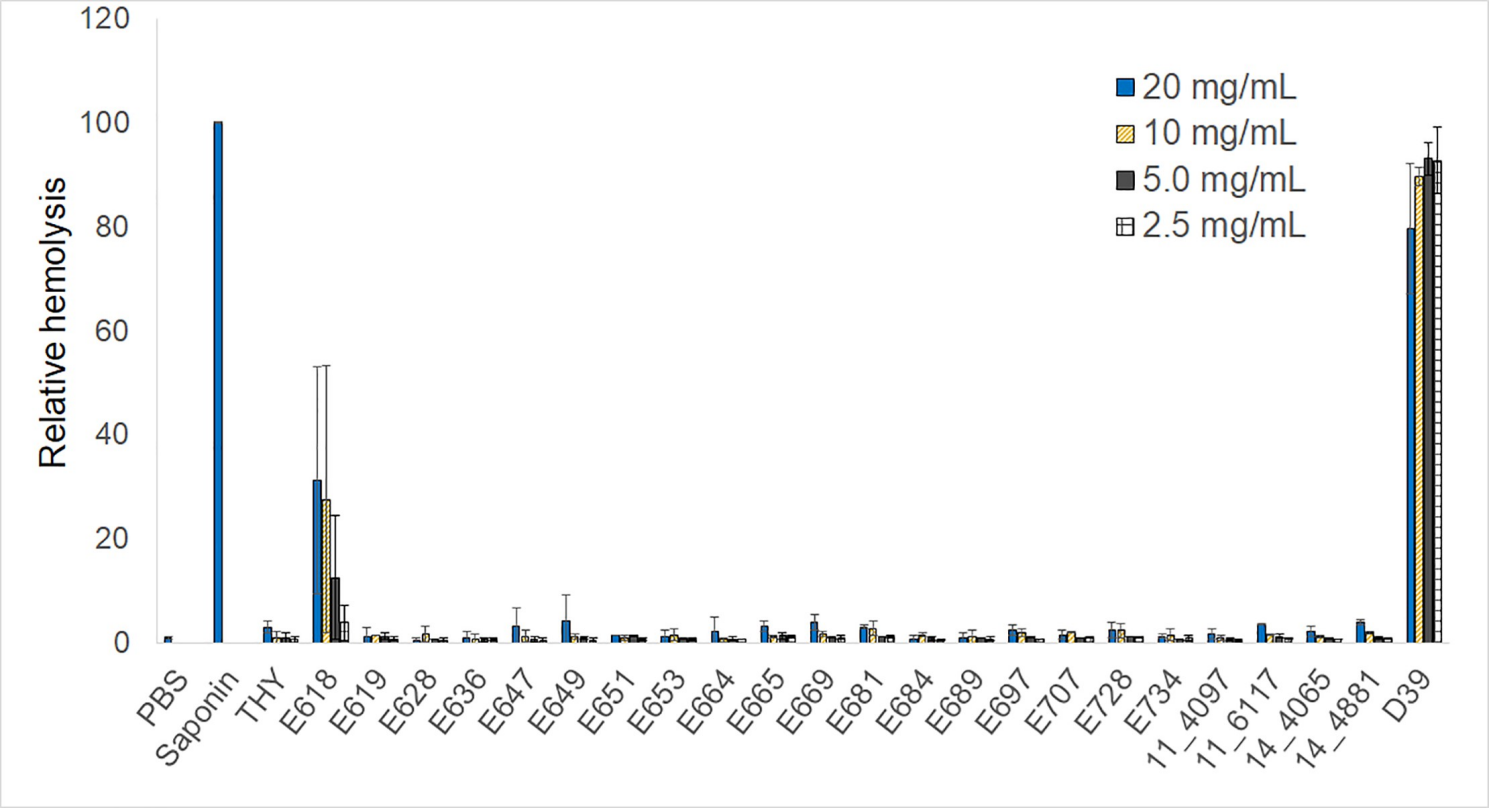
Relative mean hemolysis of sheep erythrocytes following exposure to bacterial supernatants. Each supernatant at the indicated concentrations, in addition to PBS, THY, and saponin, is listed on the x-axis. The y-axis indicates relative viability after adjustment to a scale of 100 as described in the materials and methods. Error bars indicate standard deviation. Indicators of significant differences (described in the results) are not depicted to enhance figure clarity. Data represent the averages obtained from at least 2 independent experiments.

Lysis of sheep erythrocytes by secreted material from each strain was tested for comparison to cytotoxicity of ARPE-19 cells since bacterial toxins, such as pneumolysin from *S*. *pneumoniae* and mitilysin from *S*. *mitis*, are potent hemolysins. One-way analysis of variance of controls and all samples at all concentrations indicated overall significance in terms of hemolysis (p = 0.01; Fig 2). Pairwise comparisons between each sample and PBS, saponin, and *S*. *pneumoniae* D39 determined the following: 1) THY did not cause hemolysis at any of the concentrations tested (p > 0.23 compared to PBS, p < 0.01 compared to saponin); 2) D39 was the only sample that was not significantly different than saponin in relative hemolysis, which was true for all 4 concentrations tested (p > 0.07); 3) D39 was not significantly different from PBS or the viridans samples at 20 mg/mL (p > 0.05), but was significantly different from PBS and all of the viridans samples at the 3 lower concentrations (p < 0.04) except for E618 at 10 mg/mL (p = 0.05); 4) The viridans samples were predominantly not significantly different than PBS at any of the 4 concentrations, except for 4 strains at 20 mg/mL, 2 strains at 10 mg/mL, 2 strains at 5 mg/mL, and 4 strains at 2.5 mg/mL. E618 had hemolytic activity at approximately 31%, 27%, 12%, and 4% of that of saponin at the 4 concentrations, but these quantities were not significantly different than that of PBS (p > 0.14).

### Pneumolysin gene detection

Bacterial genomic DNA from each of the 22 VGS isolates was tested for the presence of the gene encoding pneumolysin, *ply*. This 1416 base pair gene is present in *S*. *pneumoniae* and encodes a cytotoxin involved in virulence [22]. This gene has been shown to be present in some strains of VGS and primers specific for the *S*. *pneumoniae ply* gene can amplify the expected size PCR product in 13.7% of *S*. *mitis* strains [23]. We did not detect a PCR product of the expected size for any of the strains in the current study except for the positive control, *S*. *pneumoniae* D39 (not shown).

### Protease activity

**Fig 3:**
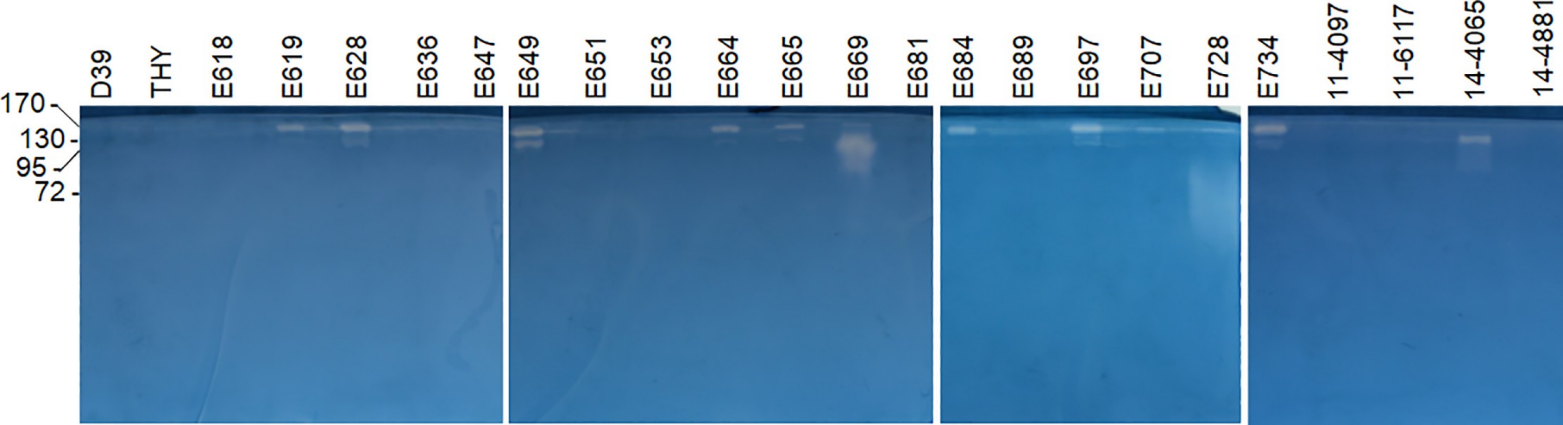
Gelatin zymography of VGS supernatants and controls. Each concentration-normalized sample was subjected to zymography to detect protease activity. Zones of clearing indicate protease activity. Molecular mass standards in kDa are indicated to the left of the gels. Two independent experiments were performed, yielding the same results. Shown are zymograms from one set of biological replicates. Each set of biological replicates was electrophoresed simultaneously using 4 zymogram gels with a lane space between each sample.

Bacterial proteases that have been implicated in the pathogenesis of ocular infections are usually secreted [25,29–31]. The extracellular milieu of each strain, therefore, was tested for protease activity by gelatin zymography. *S*. *pneumoniae* D39 and THY were negative for proteases (Fig 3). In contrast, the following 12 strains were positive: E619, E628, E649, E664, E665, E669, E684, E697, E707, E728, E734, and 14–4065. Zymography of a second set of independent biological replicates produced the same protease profiles (S1 Fig). The diffuse band for E728 that migrated to a molecular mass of approximately 72 kDa was considered a protease and not an artifact because the 2 sets of biological replicates produced the same diffuse band. Ten of the protease-positive strains were *S*. *mitis/oralis*; however, not all of the *S*. *mitis/oralis* strains (15 in total) produced protease.

## Discussion

The majority of the strains in the current study were identified as *S*. *mitis*/*oralis*, species that represent normal flora in the oral cavity. Other common oral species were also identified, but one surprising species identification was that of *S*. *pseudoporcinus*. Oral streptococci are presumed to enter the eye during surgery or intraocular injections of medication, which is the reason why ophthalmologists refrain from speech or wear face masks during these procedures [19,32–34]. *S*. *pseudoporcinus*, however, has not been documented to reside in the mouth, although one isolate was derived from a wound in a patient’s sinus cavity [35]. Little is known regarding *S*. *pseudoporcinus* other than that this species to date has been isolated predominantly from the female genital tract. *S*. *pseudoporcinus* can also be mistaken for group B streptococci due to type B serum cross-reactivity, and does share sequence homology with *S*. *porcinus*, a porcine pathogen [35–40]. The current identification of *S*. *pseudoporcinus* as an endophthalmitis isolate appears to be the first documented case. The other species identified in the current study have been reported previously, but species-level identification of VGS in endophthalmitis is lagging behind the same level of identification for other bacterial genera. Two species-level identification studies of VGS included 12 isolates comprising 7 distinct species of VGS [41] and 40 VGS isolates comprising 7 species [12].

Antibiotic susceptibilities were examined following strain identification. We chose amikacin, vancomycin, and ceftazidime because they are popular antibiotics of choice for empiric treatment of bacterial endophthalmitis [14,16,42]. Vancomycin was the most effective of the 3 antibiotics with all strains exhibiting susceptibility. However, recently vancomycin has been shown to cause toxicity resulting in hemorrhagic occlusive retinal vasculitis and subsequent vision loss [43]. If vancomycin use in the future decreases due to potential complications, effective choices for empiric treatment could become limited.

The sole strain of *S*. *pseudoporcinus* from our study, E618, appeared to be the only streptococcal strain other than *S*. *pneumoniae* D39 to exhibit cytotoxic activity. Contrary to previous studies of *S*. *pseudoporcinus* [36,38], E618 colonies did not produce zones of beta-hemolysis on blood agar (not shown). Whether E618 produces a cholesterol-dependent cytolysin similar to pneumolysin of *S*. *pneumoniae* remains undetermined at this point. A recent genomics-based approach revealed *S*. *pseudoporcinus* to produce an IgG-degrading cysteine protease belonging to a family of such enzymes in group B streptococci as well as in *S*. *suis* and *S*. *porcinus* [44]. We did not detect general protease activity in E618 by gelatin zymography; however, lack of detection by this assay does not exclude the possibility of IgG-degrading activity. As IgG is a constituent of the normal vitreous humor [45], and IgG specific for bacterial cell wall components (i.e., teichoic acid) has been detected in vitreous humor following staphylococcal endophthalmitis [46,47], examining potential evasion of *S*. *pseudoporcinus* or other species by IgG protease activity could identify new mechanisms of ocular bacterial pathogenesis.

Whatmore *et al*. [4] used *ply*-specific primers for detection of the pneumolysin gene in various strains of *S*. *pneumoniae*, *S*. *mitis*, and *S*. *oralis*. As expected, all of the *S*. *pneumoniae* strains tested were PCR-positive and 9 of 34 (26.5%) *S*. *mitis*/*oralis* were positive in that study. Interestingly, the *S*. *mitis*/*oralis* strains that were *ply*-positive were isolated from clinical specimens whereas those that were negative were commensals or isolates causing minor oral infections. In contrast, our collection of clinical endophthalmitis isolates largely appeared to be devoid of hemolytic activity and *ply* except for strain E618. The possibility of the remaining strains possessing a hemolysin/cytotoxin cannot be completely ruled out, as nucleotide sequence variations exist between members of the family of cholesterol-dependent cytolysins [23,48]. Moreover, experimental conditions for cytotoxicity and hemolysis assays could influence the limits of detection of a functional toxin. We tested extracellular material harvested after 16 hours of growth because we surmised that most virulence-associated toxins and proteases would be present in the medium by that time. Clearly, however, the virulence capabilities of VGS in the intraocular cavities extend beyond host cell toxicity. These capabilities could involve direct mechanisms such as increased fitness and evasion of host defenses or degradation of ocular proteins by proteases. An indirect mechanism likely includes destruction of ocular structures by the host inflammatory response itself. The host inflammatory response is known to contribute to the pathogenesis of bacterial endophthalmitis [49]; however, the question remains as to why the visual outcomes tend to be much worse when streptococci are isolated as the causes as opposed to *Staphylococcus aureus* or coagulase-negative staphylococci [6,8].

Putative virulence factors for bacterial ocular diseases other than cytotoxins are proteases. Protease activity has been implicated in *Enterococcus faecalis* endophthalmitis, including an association of secreted protease activity with poorer visual outcomes in patients [50]. These recent findings provide human relevance to a previous study that reported protease-positive *E*. *faecalis* to cause significantly more severe endophthalmitis than its protease-negative counterpart in a rabbit post-surgical model [25]. Ideally, we would have chosen to also examine clinical outcomes in the isolates acquired for the current study to determine whether association between protease activity and more severe endophthalmitis existed such as in the Todokoro study [50]. However, one of the limitations of our study was the unattainability of that particular patient information. We were only able to acquire visual outcomes for two strains (11–4097 and 11–6117). Visual outcomes for these two strains were poor (D. Miller, personal communication), yet neither of these strains produced protease. One or more as-yet-unidentified *Staphylococcus aureus* proteases have also been shown to cleave alpha-beta-crystallin, a vitreous humor component that protects retinal cells from apoptosis [26]. Collagen is also an important component of vitreous humor, and a protease capable of degrading collagen has been detected in rabbit eyes with *Bacillus cereus* endophthalmitis, but not in uninfected rabbits [24].

The limitation of the current study was the lack of availability of patient data. As stated previously, knowledge of visual outcomes from each of the 22 cases could help identify possible associations, or lack therof, between protease production and poor visual outcome. Future work will delineate whether proteases are significant factors in the pathogenesis of endophthalmitis caused by VGS. The strains included in this study were verified to be distinct strains and not replicates from one or more outbreaks with the possible exceptions of 11–4097 and 11–6117 (R. Kowalski and D. Miller, personal communications). Although 11–4097 and 11–6117 may have been associated with a single outbreak, our species identification and antibiotic susceptibility findings suggest that these strains are indeed distinct. Whole genome sequencing and analysis of these endophthalmitis strains will potentially uncover conserved genes that may aid in understanding the ocular virulence of VGS as well as targeting these species for alternative therapeutic research.

## Supporting information

S1 Fig Full-length zymograms from 2 sets of biological replicates. Zymograms a-d were electrophoresed simultaneously with one batch of concentrated supernatants. Zymograms e-h were electrophoresed simultaneously with a separate batch of concentrated supernatants (independent biological replicates). Pre-stained molecular mass standards, to the left of each gel, were cut from each gel after electrophoresis but prior to staining to maintain visibility. Photographs of the gels were taken with a Canon Rebel XSi camera while the gels were placed on a standard white light box. Photographs were saved to an HP SurfacePro 4 tablet. Photographs of zymograms e-h were cropped, resized while maintaining proportions, and placed together for final manuscript form (Fig 3) with Corel PaintShop Pro X9 and Microsoft Powerpoint software. Cropping and resizing were the only modifications to the photographs.
(PDF)

## References

[1] Doern CD, Burnham CA. It’s not easy being green: the viridans group streptococci, with a focus on pediatric clinical manifestations. J Clin Microbiol 2010;48: 3829–3835. 10.1128/JCM.01563-10 20810781 PMC3020876

[2] Facklam R. What happened to the streptococci: overview of taxonomic and nomenclature changes. Clin Microbiol Rev 2002;15: 613–630. 10.1128/CMR.15.4.613-630.2002 12364372 PMC126867

[3] Harju I, Lange C, Kostrzewa M, Maier T, Rantakokko-Jalava K, Haanperä M. Improved differentiation of *Streptococcus pneumoniae* and other *S*. *mitis* group streptococci by MALDI Biotyper using an improved MALDI Biotyper database content and a novel result interpretation algorithm. J Clin Microbiol 2017;55: 914–922. 10.1128/JCM.01990-16 28053215 PMC5328460

[4] Whatmore AM, Efstratiou A, Pickerill AP, Broughton K, Woodard G, Sturgeon D, et al Genetic relationships between clinical isolates of *Streptococcus pneumoniae*, *Streptococcus oralis*, and *Streptococcus mitis*: characterization of “atypical” pneumococci and organisms allied to *S*. *mitis* harboring *S*. *pneumoniae* virulence factor-encoding genes. Infect Immun 2000;68: 1374–1382. 10678950 10.1128/iai.68.3.1374-1382.2000 PMC97291

[5] Adam MK, Rahimy E, Brady CJ, Aronsky M, Hsu J, Spirn M. Endophthalmitis after penetrating ocular injury in the dentist’s chair. Can J Ophthalmol 2014:49: e154–156. 10.1016/j.jcjo.2014.09.008 25433754

[6] Chen E, Lin MY, Cox J, Brown DM. Endophthalmitis after intravitreal injection: the importance of viridans streptococci. Retina 2011;31: 1525–1533. 10.1097/IAE.0b013e318221594a 21878800

[7] Goldberg RA, Flynn HW Jr, Miller D, Gonzalez S, Isom RF. *Streptococcus* endophthalmitis outbreak after intravitreal injection of bevacizumab: one-year outcomes and investigative results. Ophthalmology 2013;120: 1448–1453. 10.1016/j.ophtha.2012.12.009 23453511 PMC3702685

[8] Gupta A, Orlans HO, Hornby SJ, Bowler IC. Microbiology and visual outcomes of culture-positive bacterial endophthalmitis in Oxford, UK. Graefes Arch Clin Exp Ophthalmol 2014;252: 1825–1830. 10.1007/s00417-014-2658-7 25028312

[9] Kuriyan AE, Weiss KD, Flynn HW Jr, Smiddy WE, Berrocal AM, Albini TA, et al Endophthalmitis caused by streptococcal species: clinical settings, microbiology, management, and outcomes. Am J Ophthalmol 2014;157: 774–780. 10.1016/j.ajo.2013.12.026 24418264 PMC3972252

[10] Kurniawan ED, Rocke JR, Sandhu SS, Allen PJ. Predictors of visual outcome and the role of early vitrectomy in streptococcal endophthalmitis. Clin Exp Ophthalmol. Forthcoming. 10.1111/ceo.13130 28949429

[11] Zamir E, Hemo Y, Zauberman H. Traumatic *Streptococcus viridans* endophthalmitis after penetrating ocular injury from orthodontic headgear. J Pediatr Ophthalmol Strabismus 1999;36:224–225. 10442733 10.3928/0191-3913-19990701-15

[12] Benz MS, Scott IU, Flynn HW Jr, Unonius N, Miller D. Endophthalmitis isolates and antibiotic sensitivities: a 6-year review of culture-proven cases. Am J Ophthalmol 2004;137: 38–42. 14700642 10.1016/s0002-9394(03)00896-1

[13] Chen X, Adelman RA. Microbial spectrum and resistance patterns in endophthalmitis: a 21-year (1988–2008) review in northeast United States. J Ocul Pharmacol Ther 2012;28: 329–334. 10.1089/jop.2011.0204 22506856

[14] Gentile RC, Shukla S, Shah M, Ritterband DC, Engelbert M, Davis A, et al Microbiological spectrum and antibiotic sensitivity in endophthalmitis: a 25-year review. Ophthalmology 2014;121: 1634–1642. 10.1016/j.ophtha.2014.02.001 24702755

[15] Melo GB, Bispo PJ, Yu MC, Pignatari AC, Höfling-Lima AL. Microbial profile and antibiotic susceptibility of culture-positive bacterial endophthalmitis. Eye (Lond) 2011;25: 382–387. 21336253 10.1038/eye.2010.236 PMC3171783

[16] Moloney TP, Park J. Microbiological isolates and antibiotic sensitivities in culture-proven endophthalmitis: a 15-year review. Br J Ophthalmol 2014;98: 1492–1497. 10.1136/bjophthalmol-2014-305030 24939423

[17] Durand ML. Bacterial and fungal endophthalmitis. Clin Microbiol Rev 2017;30: 597–613. 10.1128/CMR.00113-16 28356323 PMC5475221

[18] Goldberg RA, Flynn HW Jr, Isom RF, Miller D, Gonzalez S. An outbreak of *Streptococcus* endophthalmitis after intravitreal injection of bevacizumab. Am J Ophthalmol 2012;153: 204–208. 10.1016/j.ajo.2011.11.035 22264943 PMC3266537

[19] McCannel CA. Meta-analysis of endophthalmitis after intravitreal injection of anti-vascular endothelial growth factor agents: causative organisms and possible prevention strategies. Retina 2011;31: 654–661. 10.1097/IAE.0b013e31820a67e4 21330939

[20] Nakano K, Nomura R, Matsumoto M, Ooshima T. Roles of oral bacteria in cardiovascular diseases–from molecular mechanisms to clinical cases: cell-surface structures of novel serotype k *Streptococcus mutans* strains and their correlation to virulence. J Pharmacol Sci 2010;113: 120–125. 20501965 10.1254/jphs.09r24fm

[21] Deng L, Bensing BA, Thamadilok S, Yu H, Lau K, Chen X, et al Oral streptococci utilize a Siglec-like domain of serine-rich repeat adhesins to preferentially target platelet sialoglycans in human blood. PLoS Pathog 2014;10: e100540. 10.1371/journal.ppat.1004540 PMC4256463 25474103

[22] Ng EW, Samiy N, Rubins JB, Cousins FV, Ruoff KL, Baker AS, et al Implication of pneumolysin as a virulence factor in *Streptococcus pneumoniae* endophthalmitis. Retina 1997;17: 521–529. 9428015

[23] Jefferies J, Nieminen L, Kirkham LA, Johnston C, Smith A, Mitchell TJ. Identification of a secreted cholesterol-dependent cytolysin (mitilysin) from *Streptococcus mitis*. J Bacteriol 2007;189: 627–632. 10.1128/JB.01092-06 17071760 PMC1797409

[24] Beecher DJ, Olsen TW, Somers EB, Wong AC. Evidence for contribution of tripartite hemolysin BL, a phosphatidylcholine-preferring phospholipase C, and collagenase to virulence of *Bacillus cereus* endophthalmitis. Infect Immun 2000;68: 5269–5276. 10948154 10.1128/iai.68.9.5269-5276.2000 PMC101788

[25] Suzuki T, Wada T, Kozai S, Ike Y, Gilmore MS, Ohashi Y. Contribution of secreted proteases to the pathogenesis of postoperative *Enterococcus faecalis* endophthalmitis. J Cataract Refract Surg 2008;34: 1776–1784. 10.1016/j.jcrs.2008.06.033 18812133

[26] Whiston EA, Sugi N, Kamradt MC, Sack C, Heimer SR, Engelbert M, et al AlphaB-crystallin protects retinal tissue during *Staphylococcus aureus*-induced endophthalmitis. Infect Immun 2008;76: 1781–1790. 10.1128/IAI.01285-07 18227158 PMC2292870

[27] Clinical and Laboratory Standards Institute. Performance Standards for Antimicrobial Susceptibility Testing 27th ed CLSI supplement M100. Wayne, Pennsylvania: Clinical and Laboratory Standards Institute; 2017.

[28] Marquart ME, Caballero AR, Chomnawang M, Thibodeaux BA, Twining SS, O’Callaghan RJ. Identification of a novel secreted protease from *Pseudomonas aeruginosa* that causes corneal erosions. Invest Ophthalmol Vis Sci 2005;46: 3761–3768. 10.1167/iovs.04-1483 16186360

[29] Mochizuki Y, Suzuki T, Oka N, Zhang Y, Hayashi Y, Hayashi N, et al *Pseudomonas aeruginosa* MucD protease mediates keratitis by inhibiting neutrophil recruitment and promoting bacterial survival. Invest Ophthalmol Vis Sci 2014;55: 240–246. 10.1167/iovs.13-13151 24255043

[30] Shanks RM, Stella NA, Hunt KM, Brothers KM, Zhang L, Thibodeau PH. Identification of SlpB, a cytotoxic protease from *Serratia marcescens*. Infect Immun 2015;83: 2907–2916. 10.1128/IAI.03096-14 25939509 PMC4468537

[31] Tang A, Caballero AR, Marquart ME, O’Callaghan RJ. *Pseudomonas aeruginosa* small protease (PASP), a keratitis virulence factor. Invest Ophthalmol Vis Sci 2013;54: 2821–2828. 10.1167/iovs.13-11788 23548618 PMC3632270

[32] Doshi RR, Leng T, Fung AE. Reducing oral flora contamination of intravitreal injections with face mask or silence. Retina 2012;32: 473–476. 10.1097/IAE.0B013E31822C2958 22374155

[33] Garg SJ, Dollin M, Hsu J, Storey P, Vander JF. Effect of a strict “no-talking” policy during intravitreal injection on post-injection endophthalmitis. Ophthalmic Surg Lasers Imaging Retina 2015;46: 1028–1034. 10.3928/23258160-20151027-07 26599245

[34] Wen JC, McCannel CA, Mochon AB, Garner OB. Bacterial dispersal associated with speech in the setting of intravitreous injections. Arch Ophthalmol 2011;129: 1551–1554. 10.1001/archophthalmol.2011.227 21825179

[35] Shewmaker PL, Steigerwalt AG, Whitney AM, Morey RE, Graziano JC, Facklam RR, et al Evaluation of methods for identification and determination of the taxonomic status of strains belonging to the *Streptococcus porcinus*-*Streptococcus pseudoporcinus* complex isolated from animal, human, and dairy sources. J Clin Microbiol 2012;50: 3591–3597. 10.1128/JCM.01481-12 22933599 PMC3486230

[36] Bekal S, Gaudreau C, Laurence RA, Simoneau E, Raynal L. *Streptococcus pseudoporcinus* sp. nov., a novel species isolated from the genitourinary tract of women. J Clin Microbiol 2006;44: 2584–2586. 10.1128/JCM.02707-05 16825387 PMC1489492

[37] Collins MD, Farrow JAE, Katic V, Kandler O. Taxonomic studies on streptococci of serological groups E, P, U and V: description of *Streptococcus porcinus* sp. nov. Syst Appl Microbiol 1984;5: 402–413.

[38] Gaudreau C, Simoneau E, Labrecque O, Laurence RA, Laferrière C, Miller M, et al Epidemiological, biochemical and antimicrobial susceptibility characteristics of *Streptococcus pseudoporcinus* isolated in Quebec, Canada, from 1997 to 2006. J Med Microbiol 2007;56 (Pt 12): 1620–1624. 10.1099/jmm.0.47295-0 18033830

[39] Stoner KA, Rabe LK, Austin MN, Meyn LA, Hillier SL. Incidence and epidemiology of *Streptococcus pseudoporcinus* in the genital tract. J Clin Microbiol 2011;49: 883–886. 10.1128/JCM.01965-10 21191057 PMC3067687

[40] Suwantarat N, Grundy M, Rubin M, Harris R, Miller JA, Romagnoli M, et al Recognition of *Streptococcus pseudoporcinus* colonization in women as a consequence of using matrix-assisted laser desorption ionization-time of flight mass spectrometry for group B *Streptococcus* identification. J Clin Microbiol 2015;53: 3926–3930. 10.1128/JCM.02363-15 26468502 PMC4652085

[41] Chiquet C, Cornut PL, Benito Y, Thuret G, Maurin M, Lafontaine PO, et al Eubacterial PCR for bacterial detection and identification in 100 acute postcataract surgery endophthalmitis. Invest Ophthalmol Vis Sci 2008;49: 1971–1978. 10.1167/iovs.07-1377 18436828

[42] Clarke B, Williamson T, Gini G, Gupta B. Management of bacterial postoperative endophthalmitis and the role of vitrectomy. Surv Ophthalmol. Forthcoming. 10.1016/j.survophthal.2018.02.003 29453989

[43] Witkin AJ, Chang DF, Jumper JM, Charles S, Eliott D, Hoffman RS, et al Vancomycin-associated hemorrhagic occlusive retinal vasculitis: clinical characteristics of 36 eyes. Ophthalmology 2017;124: 583–595. 10.1016/j.ophtha.2016.11.042 28110950

[44] Spoerry C, Hessle P, Lewis MJ, Paton L, Woof JM, von Pawel-Rammingen U. Novel IgG-degrading enzymes of the IgdE protease family link substrate specificity to host tropism of *Streptococcus* species. PLoS ONE 2016;11: e0164809 10.1371/journal.pone.0164809 27749921 PMC5066943

[45] Kim T, Kim SJ, Kim K, Kang UB, Lee C, Park KS, et al Profiling of vitreous proteomes from proliferative diabetic retinopathy and nondiabetic patients. Proteomics 2007;7: 4203–4215. 10.1002/pmic.200700745 17955474

[46] Pleyer U, Mondino BJ, Adamu SA, Pitchekian-Halabi H, Engstrom RE, Glasgow BJ. Immune response to *Staphylococcus epidermidis*-induced endophthalmitis in a rabbit model. Invest Ophthalmol Vis Sci 1992;33: 2650–2663. 1639612

[47] Ravindranath RM, Hasan SA, Mondino BJ. Immunopathologic features of *Staphylococcus epidermidis*-induced endophthalmitis in the rat. Curr Eye Res 1997;16: 1036–1043. 9330856 10.1076/ceyr.16.10.1036.9015

[48] Johnston C, Hinds J, Smith A, van der Linden M, Van Eldere J, Mitchell TJ. Detection of large numbers of pneumococcal virulence genes in the streptococci of the mitis group. J Clin Microbiol 2010;48: 2762–2769. 10.1128/JCM.01746-09 20519466 PMC2916619

[49] Sadaka A, Durand ML, Gilmore MS. Bacterial endophthalmitis in the age of outpatient intravitreal therapies and cataract surgeries: host-microbe interactions in intraocular infection. Prog Retin Eye Res 2012;31: 316–331. 10.1016/j.preteyeres.2012.03.004 22521570 PMC3361607

[50] Todokoro D, Suzuki T, Kobayakawa S, Tomita H, Ohashi Y, Akiyama H. Postoperative *Enterococcus faecalis* endophthalmitis: virulence factors leading to poor visual outcome. Jpn J Ophthalmol 2017;61: 408–414. 10.1007/s10384-017-0527-8 28707018

