# Supplementary material for: Antibiotic susceptibility, cytotoxicity, and protease activity of viridans group streptococci causing endophthalmitis
Source: PLoS One. 2018 Dec 21;13(12):e0209849. doi: 10.1371/journal.pone.0209849 (PMC6303072; doi:10.1371/journal.pone.0209849)
Supplement: S1 Fig — Zymograms a-d were electrophoresed simultaneously with one batch of concentrated supernatants. Zymograms e-h were electrophoresed simultaneously with a separate batch of concentrated supernatants (independent biological replicates). Pre-stained molecular mass standards, to the left of each gel, were cut from each gel after electrophoresis but prior to staining to maintain visibility. Photographs of the gels were taken with a Canon Rebel XSi camera while the gels were placed on a standard white light box. Photographs were saved to an HP SurfacePro 4 tablet. Photographs of zymograms e-h were cropped, resized while maintaining proportions, and placed together for final manuscript form (Fig 3) with Corel PaintShop Pro X9 and Microsoft Powerpoint software. Cropping and resizing were the only modifications to the photographs. (PDF) [file pone.0209849.s001.pdf]

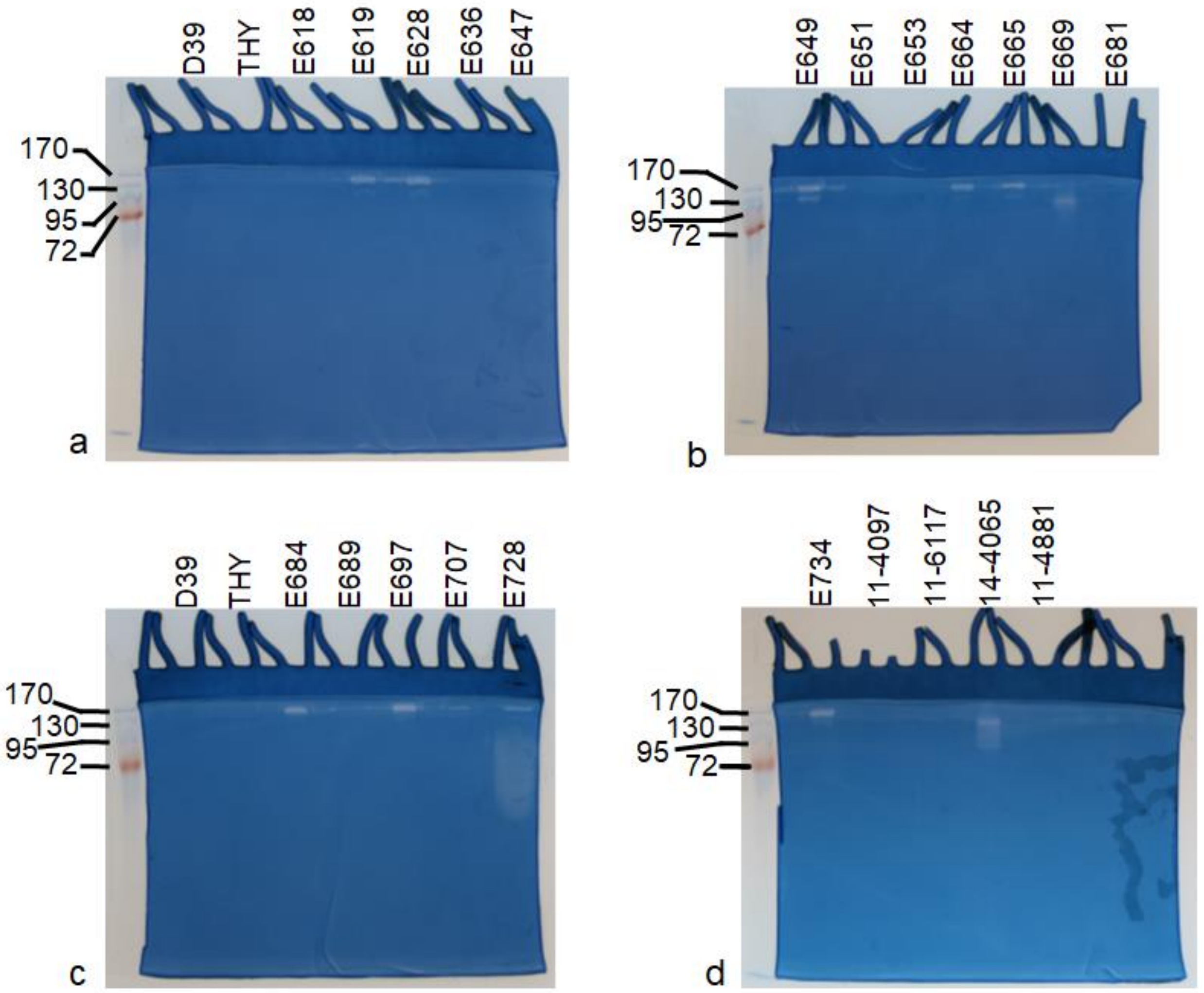
a
D39
THY
E618
E619
E628
E636
E647
170
130
95
72
b
E649
E651
E653
E664
E665
E669
E681
170
130
95
72
c
D39
THY
E684
E689
E697
E707
E728
170
130
95
72
d
E734
11-4097
11-6117
14-4065
11-4881
170
130
95
72

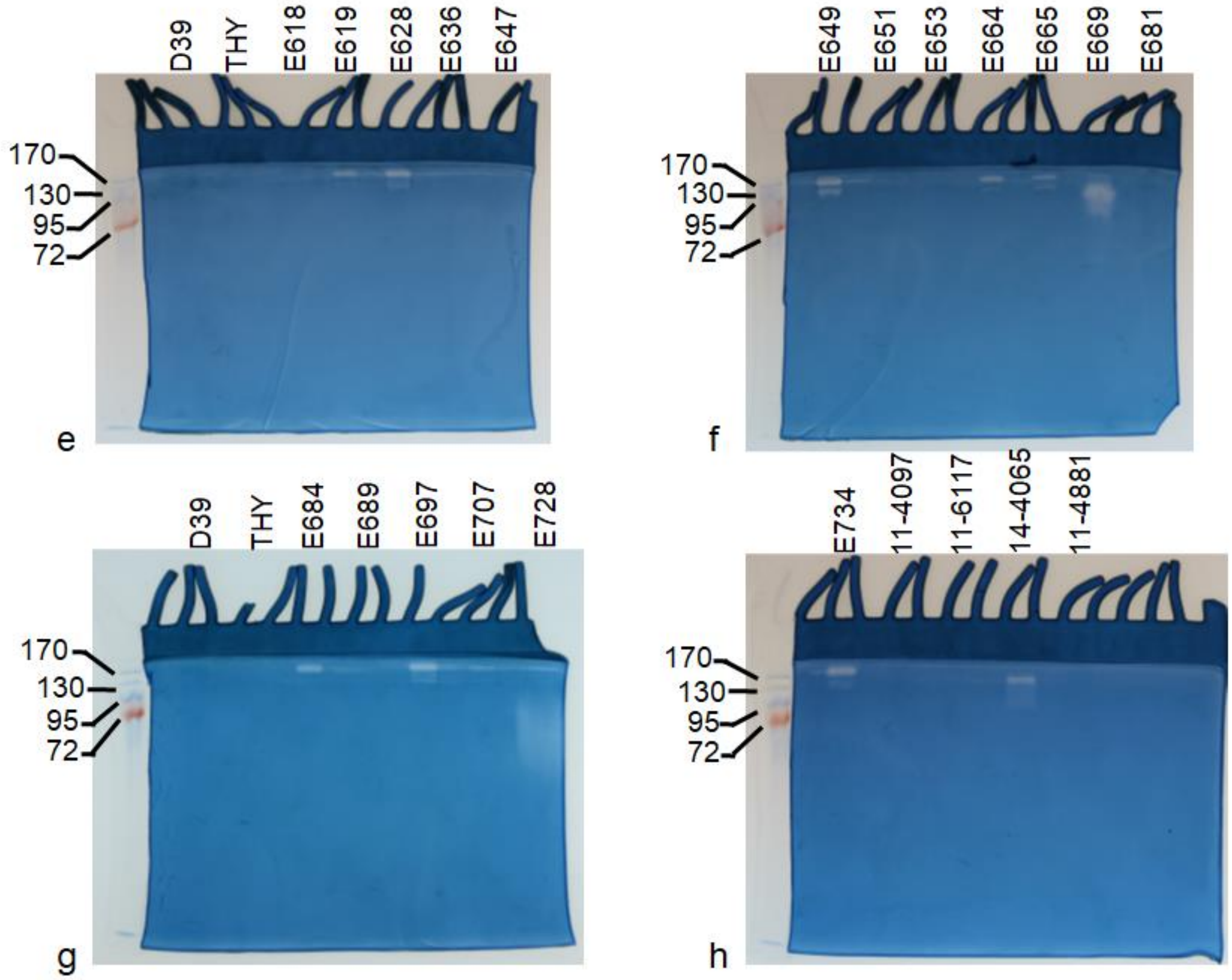

S1 Fig. Full-length zymograms from 2 sets of biological replicates. Zymograms a-d were electrophoresed simultaneously with one batch of concentrated supernatants. Zymograms e-h were electrophoresed simultaneously with a separate batch of concentrated supernatants (independent biological replicates). Pre-stained molecular mass standards, to the left of each gel, were cut from each gel after electrophoresis but prior to staining to maintain visibility. Photographs of the gels were taken with a Canon Rebel XSi camera while the gels were placed on a standard white light box. Photographs were saved to an HP SurfacePro 4 tablet. Photographs of zymograms e-h

were cropped, resized while maintaining proportions, and placed together for final manuscript form (Fig 3) with Corel PaintShop Pro X9 and Microsoft Powerpoint software. Cropping and resizing were the only modifications to the photographs.
